# Facial diplegia with hyperreflexia-a mild Guillain-Barre Syndrome variant, to treat or not to treat?

**DOI:** 10.1186/1749-7221-2-9

**Published:** 2007-04-10

**Authors:** Nitin K Sethi, Josh Torgovnick, Edward Arsura, Alissa Johnston, Elizabeth Buescher

**Affiliations:** 1 Department of Neurology, Saint Vincent's Hospital and Medical Centers, New York, USA; 2 Department of Medicine, Saint Vincent's Hospital and Medical Centers, New York, USA; 3 New York Medical College, New York, USA

## Abstract

Guillain Barre Syndrome (GBS) is readily diagnosed when the presentation is that of ascending weakness and areflexia. Atypical presentations with preserved, and at times, brisk reflexes, can be a diagnostic dilemma. We describe a patient with GBS who presented with facial diplegia and hyperreflexia on examination and discuss management options.

## Background

Guillain-Barre syndrome (GBS) is usually easily identified with its typical presentation of ascending weakness and areflexia on examination. It may however present atypically with preserved, and at times, brisk reflexes, leading to diagnostic dilemma. A patient with isolated facial diplegia and hyperreflexia on examination is presented. During the entire hospitalization, the patient developed no motor weakness and remained ambulatory. Whether treatment is warranted for this and other milder variants of GBS is also discussed.

## Case presentation

A-29-year-old right-handed Caucasian woman, who works as a model, presented to the hospital with facial weakness. She reported that a week previously she had a sore throat and was seen by her doctor who prescribed antibiotics. Four days later she developed paraesthesias in her hands and feet along with severe myalgia (day 1 of neurological manifestation). On day 3, she noted weakness in eye closure when applying eyeliner. On Day 4, she was at an audition, and was unable to smile for the camera. Later that night, she participated in a runway show. She was able to walk in high heels without difficulty. However, concerns about her face brought her to the emergency department after the show.

At presentation, neurological examination revealed facial diplegia. She was unable to close both eyes, purse her lips or smile. Deep tendon reflexes were 3(+) throughout with flexor plantar responses. She had no weakness or sensory loss in her limbs, and there were no respiratory or autonomic features on examination. Cerebrospinal fluid (CSF) showed two lymphocytes with a protein level of 162 mg/dL and normal glucose. Nerve conduction study done on Day 6 showed partial denervation of facial nerves with compound muscle actions potentials markedly decreased bilaterally. No response could be obtained on blink reflex studies bilaterally. There was no evidence of demyelination in the limbs; F waves were present with no delay in latency [Table 1, 2]. Lyme serology was negative, serum and CSF angiotensin converting enzyme levels were 10 U/L (normal, 8–52 U/L). Tests for CSF VDRL and HIV were non-reactive. Antiganglioside antibodies were not sent and no imaging studies of the brain were carried out as her presentation was consistent with a demyelinating peripheral neuropathy. The physician on hospital service elected to treat her with IV immunoglobulin (IVIG 400 mg/kg/day) for five days. By the time above treatment was initiated (Day 7) her paraesthesias had already resolved. During her entire hospitalization, she developed no motor weakness and remained ambulatory. At the time of her discharge on Day 12 she showed some improvement in her facial weakness and was able to approximate her lips as well as furrow her eyebrows. Follow up nerve conduction studies were not carried out. When last seen 6 weeks after her first presentation, she was able to smile normally and no facial weakness was evident on examination. Her deep tendon reflexes were 1(+) bilaterally.

**Table 1:** Motor and Sensory Nerve Conductions

Nerve/Site	Latency	Amplitude	Velocity
**Median nerve **(APB) Right			
Med Wrist	3.6 ms	11.58 mV	
Med Elbow	8.2 ms	9.857 mV	52 m/s
**Tibial nerve **(AH) Right			
**Med Mall**	7.0 ms	9.479 mV	
**Pop fossa**	17.0 ms	6.315 mV	41 m/s
**Facial nerve^®^**			
**Orb. Oculi^®^**	no response		
**Nasalis^®^**	2.7 ms	0.194 mV	
**Facial nerve (L)**			
**Orb oculi (L)**	no response		
**Nasalis (L)**	2.4 ms	0.465 mV	
**Median sensory**	3.5 ms		56 m/s
**Ulnar sensory**	3.2 ms		54 m/s
**Sural sensory**	4.0 ms		44 m/s

**Table 2:** F waves

Nerve	Minimum Latency
**Med nerve^®^**	28.5 ms
**Tibial nerve^®^**	54.3 ms

## Discussion

Facial diplegia has a number of causes including Bell's palsy, sarcoidosis (Uveo-parotid fever or Heerfordt Syndrome), Lyme disease, Hansen's disease (leprosy), diabetes, brainstem encephalitis, brainstem stroke, herpes zoster (Ramsay Hunt and Mekelson Rosenthal Syndrome), HIV and GBS. Isolated facial diplegia with minimal to no motor limb weakness has been described as a GBS variant [1,2]. Usually in these cases areflexia helps in distinguishing GBS as the underlying etiology. Hyperreflexia as a variant in GBS has also been described and is currently not thought to be inconsistent with the diagnosis. It is thought to be due to increased motor neuron excitability and spinal inhibitory interneuron dysfunction as evidenced by increased soleus H/M ratios and abnormal appearance of H reflexes in the small muscles of the hands and feet in some patients [3,4,6]. Hyperreflexia in GBS patients has been associated with a milder degree of peak disability, as is seen in this patient [6].

Our patient presented with isolated facial diplegia. The fact that she was able to catwalk down a runaway in high heels clearly argued against any lower limb weakness at presentation. It is unclear however if GBS patients with isolated facial diplegia warrant treatment or not. The unpredictability of the early clinical course of GBS makes it difficult to judge which patient shall worsen as the disease runs its course. Treating all these "mild" cases may risk exposing patients to the potential side effects of IVIG and plasmapheresis. There is also anecdotal evidence that transient improvement in power or paraesthesias followed by worsening may occur in relation to immune treatment i.e. immune treatment itself may predispose a patient to relapse [4,5]. In our case the treating physician who first saw her at presentation to the hospital elected to use IVIG. By the time treatment was initiated and we were involved in her care the neurological syndrome had already started to resolve as evidenced by the disappearance of paraesthesias, hence it can be debated if the patient's clinical outcome would have been any different had treatment been withheld. In their article Green et.al mention that treatment may be unnecessary in patients who remain ambulatory during the second week of illness [5]. Observation until the eight day though is advisable to be certain that the disease does not progress or relapse.

## Conclusion

It is not our intention by highlighting this case to discuss the physiology behind brisk reflexes in GBS but rather to raise the argument for withholding immunotherapy in isolated facial diplegia variant of GBS until the eighth day or so before committing these "mild" GBS patients, who are still able to walk, to IVIG or plasmapheresis.

## Competing interests

The author(s) declare that they have no competing interests.

## Authors' contributions

All authors read and approved the final manuscript.
